# Choriocarcinoma masquerading as lumbar spinal tumor: Case report and literature review

**DOI:** 10.1097/MD.0000000000032742

**Published:** 2023-01-27

**Authors:** Yitong Liu, Chan Li, Haiyan Sun, Fuli Kang, Zhenhong Zhang, Chen Yue

**Affiliations:** a Department of Gynecology and Obstetrics, The Second Hospital of Dalian Medical University, Dalian, Liaoning, China; b Department of Anatomy, Dalian Medical University, Dalian, Liaoning, China; c Department of Medical Equipment, The Third Medical University, Dalian, Liaoning, China.

**Keywords:** choriocarcinoma, lumbar spinal, metastasis

## Abstract

Choriocarcinoma is a highly invasive gestational trophoblastic neoplasm, usually metastasis to lung and brain, but occurrence of choriocarcinoma following spontaneous abortion presenting as a vertebral tumor is extremely rare, to the best of our knowledge. Because of the poor diagnosis and high malignancy, the low progression-free survival follows up.

**Patient concerns:**

We here are reporting a case of choriocarcinoma that presented with vertebral tumor induced paralysis of limbs and incontinence of urine.

**Diagnosis:**

Combined with the childbearing history, high β-human chorionic gonadotrophinin levels, and imaging examination, a clinical diagnosis was made exactly. Till the pathological results after the operation of lumbar spinal canal tumorectomy, the diagnosis was exactly clear.

**Interventions:**

After performing the laminectomy, the fierce bleeding follows up, just did the temporary limited decompression. Because of the vertebral artery embolization, lumbar spinal canal tumorectomy, spinal canal and root canal decompression, subdural decompression and hematoma removal were performed.

**Outcomes:**

After performing the operation and chemotherapy timely and positively, the patient lost consciousness and died due to the pulmonary embolism at last.

**Lessons:**

This is the first case report describing choriocarcinoma with metastases to the spine amongst Chinese population as well. Early metastasis is one of the marked tendencies of choriocarcinoma, but spine metastasis and the related spinal oppressional symptoms were found instead of vaginal bleeding in this case, which is indeed rare.

## 1. Introduction

Gestational trophoblastic disease (GTD) is derived from gestational trophoblastic epithelium of the placenta, featured with a special tumor marker β-human chorionic gonadotrophinin (HCG). Conventionally, GTDs are classified into the following groups on the basis of their histopathologic, cytogenetic and clinical features. These groups are: gestational trophoblastic neoplasm (GTN), which includes choriocarcinoma, placental trophoblastic tumor, and epithelioid trophoblastic tumor; hydatidiform mole pregnancy, which includes complete hydatidiform mole, partial hydatidiform mole, and aggressive hydatidiform mole; non-neoplastic lesions, which include abnormal placental site reactions and placental nodules; and abnormal (non-hydatidiform) villi lesions.^[1,2]^ Each has a distinct pathobiology attributing to the proliferative ability of its constituent, although it is a rare and highly malignant tumor which is known for its high β-HCG levels, rapid metastasis to multiple organs such as brain and lung and is sensitive to chemotherapy. Secondary to normal or abnormal pregnancy, it is termed as pregnant choriocarcinoma. This disease mainly occurs in childbearing women and is caused by the malignance of the gestational trophoblastic cells.^[3,4]^ Moreover, choriocarcinoma has a tendency of early metastasis through blood-borne dissemination. 30% of the patients showed metastasis when diagnosed. The most common sites of metastasis are lungs (94% of choriocarcinoma cases with metastasis), followed by the vagina (44%), liver (28%) and brain (28%), and skin, gastrointestinal organ, kidney, breast, and bone. According to the variety of metastatic sites, the clinical manifestations also show significant differences, and the symptoms are mostly similar to the primary tumors at the metastatic sites.

## 2. Case report

A 43-year-old female with the main complaint of 1-month lasting severe pain in left leg and lumbago, accompanied with urinary and fecal incontinence, was admitted to emergency in the Second Hospital of Dalian Medical University (Dalian, China) in January 2021.

**Figure 1.**
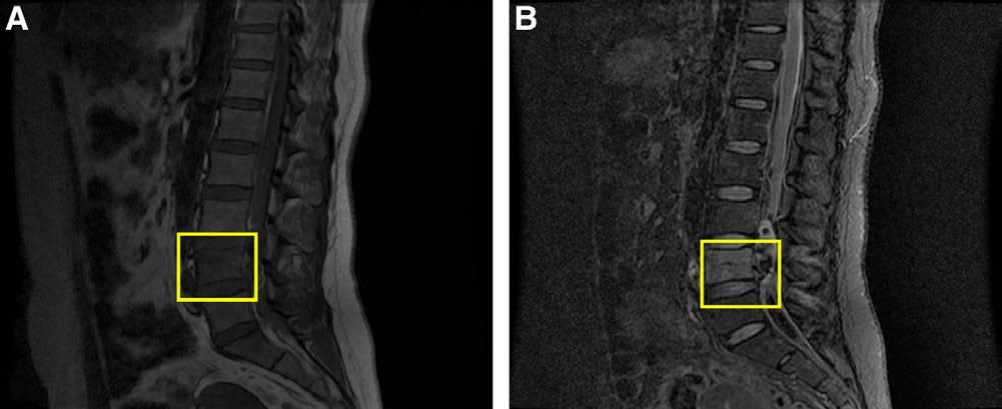
Magnetic resonance image (MRI) of the spine. (A) Sagittal T1-weighted image of the spine. (B) Sagittal T2-weighted image illustrated high signal of lumbar 4 vertebral body, which suggested spinal canal mass occupation on L4.

Judging from the imaging features, it was resembled an aggressive mass lesion, with enhancement and mass occupying effect. Lumbar spine magnetic resonance imaging demonstrated high signal in T2-weighted images of lumbar 4 vertebral body, which suggested spinal canal mass occupation (Fig 1). According to her medical history, this patient had irregular menstruation and the last period was unknown. Serum test showed that the β-HCG was 8511 mIU/mL; based on the results of transvaginal ultrasound, no obvious gestational sac was observed in the uterus, and no abnormalities were found in bilateral adnexal regions. Moreover, the patient acclaimed spontaneous abortion in the past; and the β-HCG value ever reached to about 2000.00 mIU/mL in May 2019 then went down to about 20.00 mIU/mL; the follow-up value of the β-HCG was missed because of the recovery of menstruation. Collectively, it was suggested ectopic pregnancy, the possibility of trophoblastic tumor was not excluded. The changes of the β-HCG and gynecological ultrasound should be monitored. A clinical diagnosis of lumbar tumor was made exactly.

**Figure 2.**
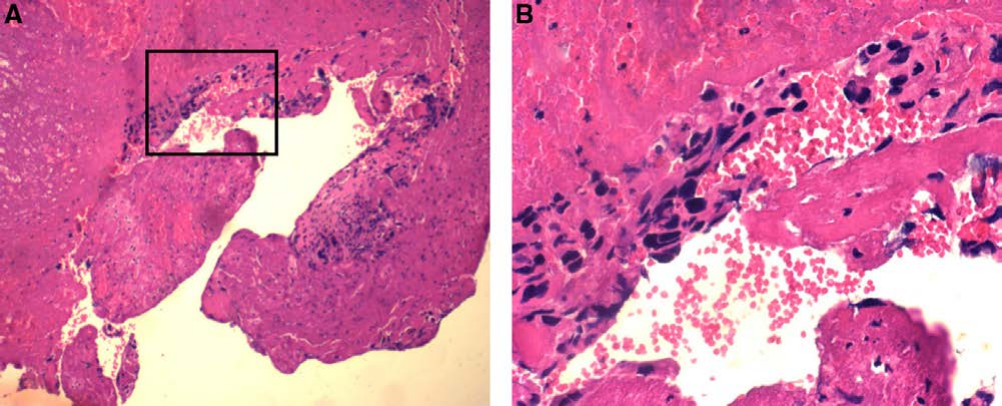
Hematoxylin and eosin stain of the intravertebral mass. Cellular heteromorphism, partially resemble syncytiotrophoblasts, no chorionic villi are seen. (A) Low-power view ×100. (B) High-power view ×400.

**Figure 3.**
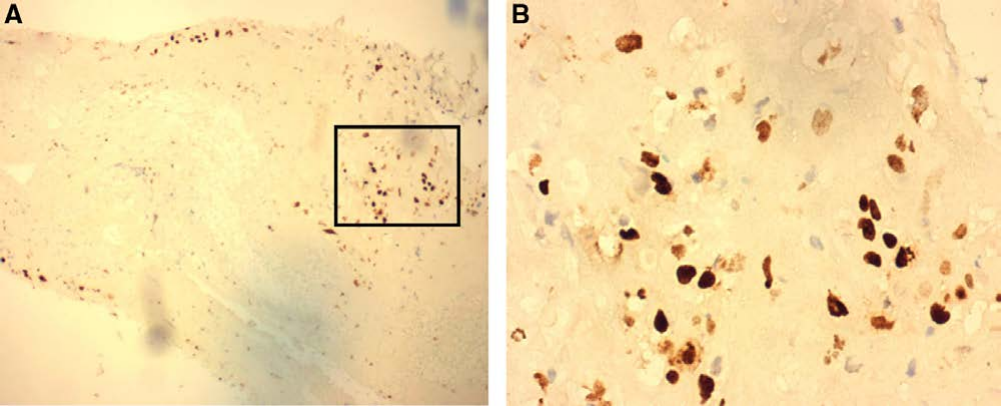
Immunohistochemical stain: trophoblast-like cells showing positive staining for Ki-67. (A) Low-power view ×100. (B) High-power view ×400.

**Figure 4.**
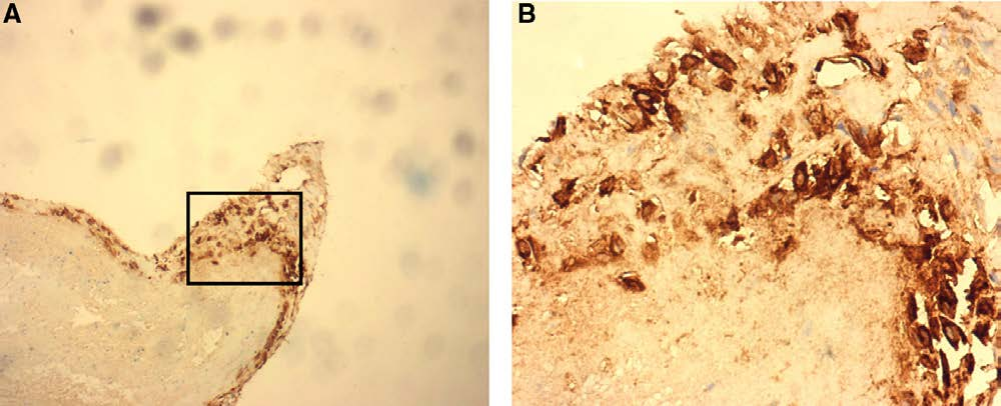
Immunohistochemical stain: trophoblast-like cells showing positive staining for hCG (human chorionic gonadotrophin). (A) Low-power view ×100. (B) High-power view ×400.

**Figure 5.**
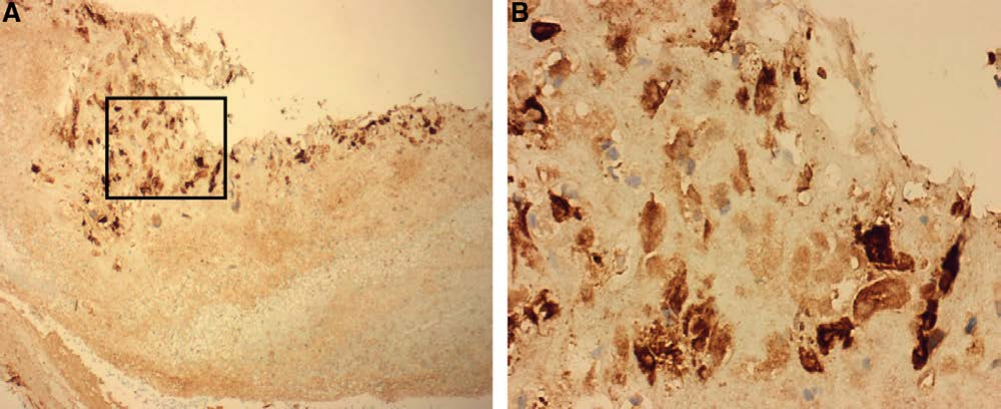
Immunohistochemical stain: trophoblast-like cells showing positive staining for AE1/AE3. (A) Low-power view ×100. (B) High-power view ×400.

The patient was proposed to perform the laminectomy, decompression of L3 to 4 and internal fixation with pedicle screw of L3 to 5. Due to the fierce bleeding, just did the temporary limited decompression. In January 10, 2021, after the L4 vertebral artery embolization, lumbar spinal canal tumorectomy, spinal canal and root canal decompression, subdural decompression and hematoma removal were performed. The pathological result suggested that: trophoblastic tumor, tending to choriocarcinoma (Figs. 2–5). At the same time, the blood β-HCG value was 5630 mIU/mL.

As a result, the clinical diagnosis of choriocarcinoma (stage IV, high risk) was confirmed. The patient underwent chemotherapy of 3 cycles of EMA-CO (day 1: etoposide/actinomycin-D/methotrexate; day 2: etoposide/actinomycin-D; day 8: vincristine/cyclophosphamide) on February 19, 2021, March 7, 2021, and April 2, 2021. During the chemotherapy, the patient developed grade 3 granulocytopenia and gastrointestinal reaction and was treated with symptomatic leukocytosis and rehydration. Blood β-HCG value decreased from 7027.00 mIU/mL on February 19, 2021 to 441.90 mIU/mL on April 10, 2021, which suggested that the chemotherapy was effective. Furthermore, low back pain occurred in March 2021. Results of 3.0 magnetic resonance imaging demonstrated abnormal signal of sacral 1 and 2 vertebrae, which suggested that the mass lesion was larger than before, implying the possibility of metastasis. The blood β-HCG value was 298.30 mIU/mL on April 22, 2021 and increased to 317.90 mIU/mL on May 4, 2021, suggested a tendency of drug resistance. Following that, the patient underwent cycle 4 chemotherapy changed to EMA (day 1: etoposide/ actinomycin-D/methotrexate; day 2: etoposide/actinomycin-D) protocol, and on May 29, 2021 the chemotherapy protocol was adjusted to the FAEV (day 1: vincristine/ 5-fluorouracil/etoposide/actinomycin-D; day 2–5: 5-fluorouracil/etoposide/actinomycin-D) protocol. Computed tomography (CT) scan on June 21, 2021 demonstrated left-sided pneumothorax (about 10%); right lower lobe parapneumonic mass, possible the malignant occupancy; multiple small nodules were observed in both lungs; bilateral adrenal region occupancy, possible metastasis was suggested. On the cycle 2 FAEV protocol at July 8, 2021, a novel mass in the lumbar surgical area was found and at July 25, 2021, ultrasound suggested a mixed mass around the vertebrae at the surgical area. Blood β-HCG value was 675.30 mIU/mL at August 1, 2021, the biopsy of the mass suggested abscess. Lumbar posterior abscess incision and debridement were performed at August 8, 2021. The patient presented with symptoms of chest tightness and shortness of breath at September 30, 2021, CT scan suggested left-side pneumothorax. To release the symptoms, left thoracocentesis tube placement was performed, and the drainage tube was removed after improvement. Few days later, the patient received 2 times of right-side thoracocentesis tube placement at October 11, 2021 and November 7, 2021. Additionally, posterior lumbar debridement was performed at October 31, 2021. At 12:49 November 27, 2021, the patient suddenly lost consciousness, and was clinically determined to death due to the pulmonary embolism.

## 3. Discussion

The case we reported features novel progress and some special clinical significances, on one hand, the patient was admitted in the spine surgery department with the symptoms of limited lower limb mobility and incontinence due to the metastases of the tumor to the lumbar spine. The diagnosis was the primary vertebral canal occupancy, and thus the surgery was performed under the indicators of primary vertebral tumor. Pathological results suggested the mass was the spinal canal metastasis of the choriocarcinoma.

Choriocarcinoma has a markedly tendency of early metastasis, just like what we found in this case, although vaginal bleeding is the common symptom of choriocarcinoma.^[5]^ In this case presenting no vaginal bleeding, but spinal oppressive symptoms, the spine metastases were very rare compares to common lung metastases.^[6,7]^ Table 1 presents all cases of reported choriocarcinoma metastases to the spine up to date.^[2,6–12]^ So far, clinically, limited cases of choriocarcinoma spine metastases have been reported. Choriocarcinoma is a chemosensitive malignant cancer with a good prognosis, even in advanced stages. The prognosis of such patients could be poor as still. According to the reported cases, most patients with a systematic treatment based on multi-drug chemotherapy combining radiotherapy and surgery ultimately ended up with death.^[2,6–12]^ Some characteristic markers of the choriocarcinoma are helpful for the accurate diagnosis, Kavanagh proposed a correlation with the upregulated levels of vascular endothelial growth factor, thought to be involved in the development of trophoblastic disease.^[13]^

**Table 1:** Measurements of HCG value in chronological order.

Primary author	Age/gender	History	Time to previous pregnancy	Tumor location	Symptom	Imaging manifestation	Treatment	Outcome
Kuten, 1977	20/F	Hydatidiform mole	8 mo	T1–3 epidural, head	Back pain, paraparesis	Not mention	Surgery: laminectomy of L1–3 + resect partial epidural tumor. Chemotherapy: methotrexate. Radiotherapy to the lumbar region.	Residual foot drop, no evidence of disease at 4-yr follow-up
Eskreis,1988	33/F	hydatidiform mole	30 mo	T2–3 epidural, stomach, head	Fatigue, melena, syncope, pain, L2 sensory loss	CT: a contrast-enhancing lesion in the left occipital lobe. Myelography: a high grade epidural block at L2–L3.	Surgery: L2–3 laminectomy. Chemotherapy:methotrexate + actinomycin D + cytoxan	Full strength and continence resumed, alive at 6 wk post-op
Rustin,1989	F	Not mention	30 mo	Lumbar spine	Not mention	Not mention	Chemotherapy: EMA-CO	Not mention
R.Vami,1993	27/F	Normal-term dilivery	4 yr	S5, left gluteal region lung	Limp, extreme pallor, backache and pain in legs. Menorrhagia, breathlessness, hemoptysis	X-ray: osteolytic lesion, chest: cannonball	Chemotherapy:12 cycles (methotrexate, actinomycin-D and chlorambucil) Radiotherapy: sacrum	Lung metastasis disappear, β-HCG normal
Williamson, 1994	26/M	Cryptorchidism, torsion of the right testis	7 yr	Vertibral bodies (autopsy), testis, liver, spleen, kidney, pancreas, thyroid, adrenals, eyes, lung	Loss of vision, chest pain, dyspnea	X-ray: chest-a large right pneumothorax with collapse, and a number of cyst-like radio lucencies near the edge of the right lower lobe.	Radiotherapy and chemotherapy to both orbits	Remission at 4-yr follow-up
Beskonakli, 1998	44/F	Uterine carcinoma (total hysterectomy)	2 yr	T5 epidural, uterus	L2 sensory loss, paraplegia, loss of all deep tendon reflexes below the level of T6, lower extremity, weakness, urinary incontinence	X-ray: a complete block with intact bony structures at T5 level.	Surgery:laminectomy at T 4–6 level chemotherapy (blackish brown in color, was relatively soft and easily removed)	Died from bronchopneumonia 5 mo later
Balat, 2004	24/F	Ovarian choriocarcinoma	Not mention	T3–5 epidural, T5 body, ovary, sternum	Paresthesias	MRI: a hypointense signal on T1W1 and a hyperintense signal on T2W1, a soft tissue mass in the spinal canal. CT: compression of the thecal sac and multiple homogeneous solid nodules in both lungs.	Surgery: total hysterectomy, bilateral salpingo-oophorectomy, infracolic omentectomy, and pelvic lymph node dissection + excise mid sternum + T5 corpectomy + removal the mass in the epidural space chemotherapy: BEP	Death after first chemotherapy cycle
Menegaz, 2004	45/F	Hydatidiform mole	2 yr	L2-S1 epidural, iliopsoas, lungs, uterus	Edema in the lower right limb, pain, RLE paraplegia, LLE paresis, urinary incontinence	X-ray: chest-nodular opacity compatible with pulmonary metastases. US: uterus volume enlarge, heterogeneous echo texture, arteriovenous fistulae	Chemotherapy: 7 EMA-CO. Radiotherapy: 21 sessions	Death from sepsis caused by febrile neutro-penia
Natio et al, 2009	38/F	Normal-term delivery	8 mo	T2 body, lung	Back pain, vertebral knock pain, no sensory and motor disturbances, hemoptysis	MRI: low intensity on T1W1 and intermediate-to-high intensity on T2W2 weighted, and showed faint enhancement on contrast-enhanced T1W1, an abnormal engorgement of the epidural venous plexus in the anterior epidural space slightly compressed the spinal cord.	Surgery: L2 vertebrectomy, L1–3 posterolateral fusion. Chemotherapy: 3 methotrexat. Radiation: lumbar	Died from respiratory failure 87 d after the second operation
Lee, 2010	33/F	Cesarean due to intrauterine fetal death	2 wk	L3 body, pedical with epidural extension, brain, lung, uterus	Headache, dyspnea, hemoptysis, backpain, progressive paraparesis	CT: chest-2 cm sized nodular mass lesion, hemorrhage, hypervascularity brain-hemorrhagic, highly attenuated, subtle, peripheral enhancing nodule with peripheral edema	Chemotherapy: EMA-CO	Shrinking epidural mass, ambulatory normal β-HCG
Guber, 2011	26/M	Testicular tumor	4 mo	Spine, eye, lung, brain, kidney, liver	Loss of vision, a non-pigmented tumor	Not mention	Surgery: left radical orchiectomy. Chemotherapy: 4 BEP + radio-therapy on eyes, spine, head	Remission at 4 yr follow-up
Ko, 2012	21/F	Comlete hydatidiform mole	10 mo	L2 body, thoracic, intramedullary, brain, lung	Headache, nausea, altered mentation, visual field defect, paraplegia and sensory loss below the nipples	CT: brain-a hematoma in the left temporoparietal region, hemorrhage in the right frontal region. Lung-multisized multiple nodules. MRI: a rimenhancing mass in the L2 vertebral body	Surgery: Craniotomy, removal hematoma with the nodular mass in the left temporo-parietal region. Radiotherapy and multiagent chemotherapy	Died from respiratory failure 13 mo after the diagnosis
Sarika Singh, 2014	24/F	Normal-term dilivery	4 mo	Bone marrow	Fever, oliguria, vomiting, fainting, and giddiness	diffuse hypoechoic mass in the liver	No	Disease
Schoch J,et al, 2014	30/M	Testicular mass	3 yr	L2–3, liver, lung, pelvic, head	Increasing low back pain, lower extremity weakness, paresthesias, poor appetite	MRI: a 12 cm left-sided retroperitoneal mass that extended into the spinal canal with severe thecal sac compression at the L2 level. Scrotal ultrasound: an echogenic region within the left testis with clustered microlithiasis suspicious for primary testicular neoplasm.	Surgery: Laminectomies + posterolateral pedicle screw fusion. Chemotherapy: etoposide + ifosfamide + cisplatin.	Death on postoperative day 21.
Xiaoman Li, 2015	68/F	Normal-term dilivery	42 yr	Scalp, lung, scapula, atlantoaxial joint skull, neck, and lymph nodes	Cough, expectoration, headaches anorexia	CT: a number of nodular opacities with blurred edges in the right upper lobe of the lung, The largest opacity measured 3.6 × 3.7 cm, and exhibited focal cavitation	Chemotherapy: comprising tegafur (800 mg) and actinomycin D (200 µg), partial injected with metho-trexate (20 mg).	Succumbed to infection
Hashermi SM,et al, 2016	32/F	Ectopic pregnency	60 d	T5–T7, lung, liver, uterus	Paraplegia, urinary retension	MRI: heterogeneous paravertebral mass Bus: heterogeneous highly vascular ill-defined area	Chemotherapy: 4 EMA-CO	Lung metastasis disappear, β-HCG normal
Tatsuya,Ishigura,et al, 2017	41/F	Missed abortion	7 mo	L3–5 lumbar spine, cervical, lungs, liver, spleen, brain	Lumbago and leg paralysis, headache, dyspnea	MRI: an intense high-signal lesion in the L4 CT: multiple metastases to the brain, lungs, liver, and spleen	Surgery: Laminectomy (L3/4.4/5) and tumorectomy around the epidural cavity + cervical tumor removed. Chemotherapy: EMA-CO	Died of respiratory failure
Yuan-Yuan Lin et al, 2020	40/F	Caesarean	10 mo	Th5–6, Th8–12, L1 and S2, Th9–L1 liver, lungs	Cough, hemoptys, lower back pain, hemoptysis	CT: revealed nodular opacity lesions distributed in both lungs ultrasonography: revealed multiple masses in the liver endometrium (−)	Surgery: CT-mediated percutaneous radiofrequency ablation of the right liver. Chemotherapy: EMA-CO(6) + FAEV (1), EMA-EP(8) Radiotherapy:18	hCG normal 1 yr follow up alive

BEP = bleomycin, etoposide, cisplatin, CT = computed tomography, EMA-CO = etoposide, methotrexate, actinomycin-D, cyclophosphamide; FAEV = 5-fluorouracil, actinomycin-D, etoposide, vincristine, HCG = human chorionic gonadotrophinin, MRI = magnetic resonance imaging, US = ultrasonography.

The typical imaging findings are dependent on the site of metastasis, which is associated with extensive bleeding. In lung, metastatic nodules are showing ground-glass attenuation (CT halo sign) which is consistent with hemorrhage. As bleeding extension involving to diffuse alveolar hemorrhage, the opacities may emerge throughout the lung.^[14]^ In some cases, metastases may produce pseudoaneurysm or arteriovenous fistulas, which differential diagnosis from renal cell carcinoma, thyroid carcinoma, angiosarcoma, or hemangioendothelioma.^[15]^ Childbearing history and rising β-HCG could help to identify the disease. Nowadays deoxyribonucleic acid geno-typing using polymerase chain reaction to analyze short-tandem repeat polymorphism has emerged as a powerful tool in the precise diagnosis and classification of GTDs. In this case, the patient was characterized by urinary and fecal incontinence, impaired mobility caused by the metastasis to the spine, accompanied with adrenal metastasis, and multiple organs metastases, diagnosed by operation pathology.

**Figure 6.**
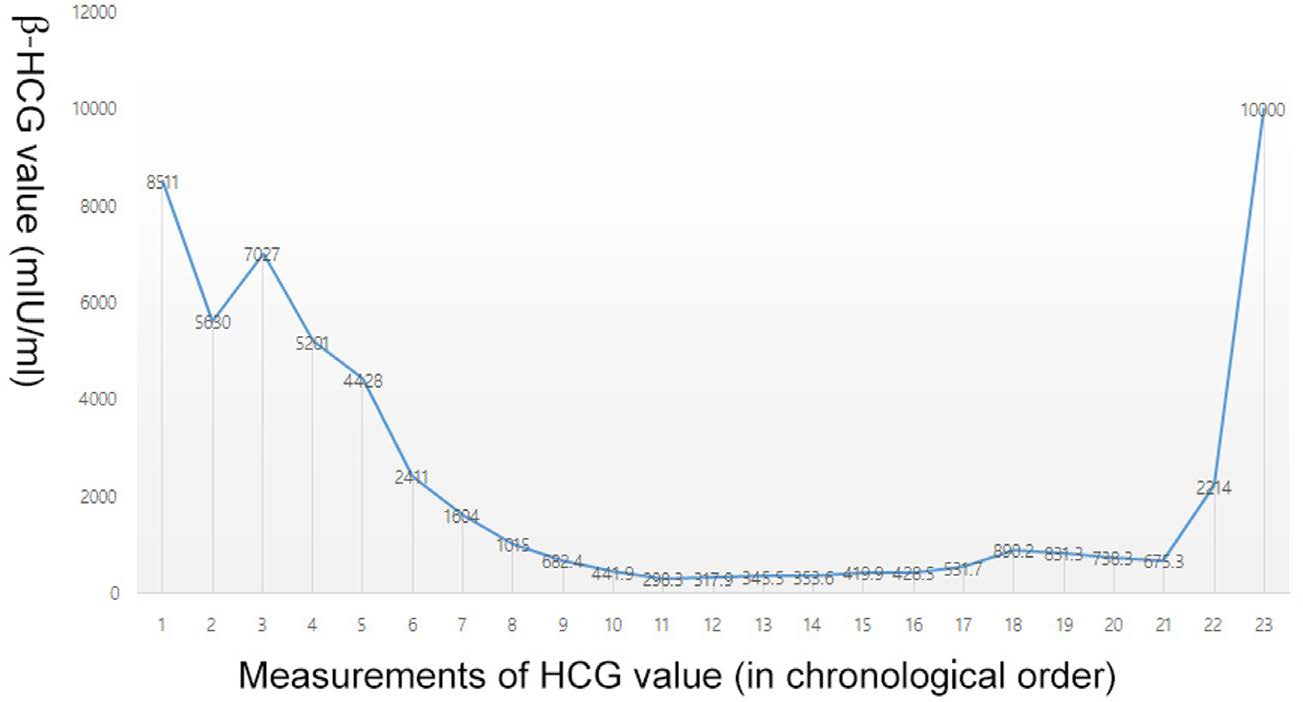
Changes of β-HCG value in chronological order.

According to the Federation International of Gynecology and Obstetrics prognostic score standard, the choriocarcinoma of this patient is classified as a high-risk type, thus poor prognosis could be estimated. Initially the patient underwent EMA-CO chemotherapy. After the patient received 3 cycles of chemotherapy, the blood β-HCG showed a downward trend, indicating that the chemotherapy was effective. However, due to the severe bone marrow suppression and gastrointestinal side effects, the treat plan was adjusted to EMA protocol. Unexpectedly, the patient showed further tumor growth and metastasis, as well as the limited mobility of both lower limbs, and the decrease of blood β-HCG level was not ideal, implying the tendency of drug tolerance. As a result, the chemotherapy was adjusted to the FAEV regimen. For this time, the patient could not tolerate the chemotherapy, so the chemotherapy was not complete. Simultaneously, the patient’s blood β-HCG level did not drop but increased (Fig. 6). During the third FAEV chemotherapy, the patient showed extensive metastasis throughout the body, multiple pneumothoraxes, as well as infection and abscesses in the spine surgery area. Eventually, the patient died of pulmonary embolism.

In summary, the treatment principle of choriocarcinoma is mainly chemotherapy, supplemented by other treatment methods such as surgery and radiotherapy. Selection of the treatment regimen is based on the federation international of gynecology and obstetrics stage, age, fertility requirements, and economic situation.^[12]^ In general, even for the patients with high risk of metastasis, the complete remission rate and survival rate can still reach to >90%, and the complete remission rate of combined chemotherapy for high-risk and drug-resistant choriocarcinoma can also reach to >80%.

Molecular profiling suggests that strong PD-L1 expression in GTN, and anti-PD-1 or anti-PD-L1 might represent a novel treatment strategy for the management of chemoresistant GTN. PD-1 targeted therapy manifested useful in various malignant tumors, such as non-small-cell lung cancer, breast cancer, ovarian cancer, and melanoma.^[16]^ There have been some cases reported of GTN treated with pembrolizumab in literature, about 66.7% had a complete response, 25% had a partial response, and only 1 patient 8.3% had disease progression. Despite these successful cases proving the effectiveness of pembrolizumab in GTN, till nowadays no reviews have been provided the data to identify the safety of pembrolizumab in this disease setting so that pembrolizumab is not licensed for application in GTN for the moment.^[17]^

On the other hand, finally the patient died from pulmonary embolism. Patients with cancer are high-risk groups for venous thromboembolism (VTE). Studies have shown that the risk of VTE formation increased 4.1 times in cancer patients and 6.5 times in patients receiving chemotherapy. VTE is one of the most important complications of tumors and one of the causes of death of tumor patients. Collectively, choriocarcinoma patients are highly potential to develop thrombosis during chemotherapy. The reason may be related to increased level of β-HCG, by which caused side effects and increased blood coagulation. At the same time, the patient showed compression symptoms caused by lumbar spinal metastasis, restricted lower limb movement, which increased the risk of thrombosis. For the tumor patients with a Khorana score ≥2, primary prevention of VTE can be performed.^[18,19]^ If this patient is evaluated for venous thrombosis during chemotherapy, whether timely prevention will improve the patient`s prognosis should be considered. Analyzed the whole procedure of this case, the final cause of death of the patient was considered to be pulmonary embolism caused by cancer thrombus.

## 4. Conclusion

We have reported a rare case of a spinal metastasis of a choriocarcinoma masquerading as lumbar spinal tumor firstly performed in Chinese population. The patient ended up with death eventually, despite the surgery and multidrug chemotherapy. Choriocarcinoma metastasis to the spine is exceedingly rare and likely represents end-stage of the disease with poor prognosis. Early diagnosis and treatment might enable a reduction to the mortality rate. Timely prevention of VTE may improve the patients` prognosis.

## Acknowledgments

The authors thank all the other staff of the Department of Obstetrics and Gynecology, The Second Hospital of Dalian Medical University.

## Author contributions

**Conceptualization:** Yitong Liu, Chen Yue.

**Data curation:** Haiyan Sun, Fuli Kang.

**Investigation:** Yitong Liu.

**Project administration:** Chen Yue, Chan Li.

**Software:** Yitong Liu, Zhenhong Zhang.

**Supervision:** Chen Yue.

**Validation:** Chen Yue.

**Writing – original draft:** Yitong Liu, Chen Yue.

**Writing – review & editing:** Yitong Liu, Chan Li.
